# School-Based Prevention in Very Remote Settings: A Feasibility Trial of Methods and Measures for the Evaluation of a Social Emotional Learning Program for Indigenous Students in Remote Northern Australia

**DOI:** 10.3389/fpubh.2020.552878

**Published:** 2020-11-17

**Authors:** Gary William Robinson, Eunro Lee, Sven Robert Silburn, Patricia Nagel, Bernard Leckning, Richard Midford

**Affiliations:** ^1^Menzies School of Health Research, Charles Darwin University, Darwin, NT, Australia; ^2^School of Health and Biomedical Sciences, RMIT University, Melbourne, VIC, Australia; ^3^National Drug Research Institute, Curtin University, Perth, WA, Australia

**Keywords:** indigenous youth, remote communities, social emotional learning (SEL), middle schools, suicide prevention, feasibility and acceptability

## Abstract

**Purpose:** Skills for Life (SFL) is a social-emotional curriculum for Indigenous middle school students that was co-developed with educators and community members in a remote community of northern Australia. This preliminary study aimed to test the feasibility of processes and methods of data-gathering, the reliability of youth self-report measures, and to identify the direction of effects for an evaluation of a longer-term pilot of the curriculum.

**Design/Methodology/Approach:** Indigenous Students in years 7–9 of a remote school participated in SFL over 2 years. The Strengths and Difficulties Questionnaire (SDQ), Kessler 6 (K6), and a purpose-designed Connected Self Scale (CSS) were administered to 63 students pre- and post-program.

**Findings:** Only the K6, Prosocial behavior (SDQ), and two CSS subscales showed sufficient internal consistency for analysis. Change was positive but non-significant for SDQ and CSS. There was evidence of a dosage effect: students receiving the intervention over 2 years showed greater reduction in psychological distress than other students. There was no evidence of iatrogenic effects.

**Conclusions:** The feasibility pilot is a critically important phase in the development of evaluation design and cjhoice of evaluation measures for challenging remote settings. This study found that evaluation of SFL with culturally and linguistically distinct Indigenous middle school students using self-report measures is feasible. However, the SDQ may not be suitable for this project. High levels of psychological distress suggest the need to investigate sources of life stress and potential supports for adolescent resilience in this context. This preliminary pilot aimed to trial methods and measures for evaluation of a social-emotional curriculum developed specifically for remote Australian Indigenous students who are at risk of poor psychosocial outcomes. No studies have examined the appropriateness of standardized self-report measures for evaluation of SEL with this student population in remote school settings.

## Introduction

The Northern Territory (NT) accounts for 17.5% of Australia's landmass but is the smallest of eight Australian states and territories, with a population of 245,786 in 2018, of whom 30% are Indigenous. Suicide is the leading cause of death among NT adolescents, with by far the highest rates among Indigenous youth in rural and remote communities, exceeding those of any other Australian state or territory (1).

Aboriginal children in remote communities are severely disadvantaged in educational terms. As many as half of remote Aboriginal children are assessed as developmentally at risk or vulnerable on measures of language, cognitive and social-emotional development at school entry, while school attendance is around 60% in middle school and declines steeply in secondary school (2). According to Australia's national program of literacy assessment, years 7 and 9 Indigenous students in remote schools are in the lowest bands of literacy attainment (3). While English is the language spoken at school, most students speak languages other than English at home.

International studies have shown that school-based social-emotional learning (SEL) programs can contribute to the development of social competencies, emotional self-awareness, improved resilience, and academic learning (4). Research has shown that effective school-based SEL and mental health promotion programs can be delivered by teachers (5, 6).

However, few suicide prevention programs are curriculum-based and taught by teachers in the classroom. A review of programs found only two universal, curriculum-based interventions with evidence of effectiveness, and the majority of studies reviewed were selective and targeted programs taught by nurses, counselors, and others (7). Only a very small number of universal curriculum-based programs have demonstrated any direct impact on risk and protective factors for suicide (8, 9).

It has been argued that universal preventative programs can have unintended iatrogenic effects and that selective programs specifically targeting students at risk may be preferable (10). A review of the effectiveness of universal suicide prevention programs aiming to improve adolescent help-seeking behaviors found that help-seeking among at-risk groups actually declined after the interventions, when compared with controls (9). However, universal programs can contribute to students' acquisition of social and emotional competencies and help them to acquire an understanding of risks to their well-being. They may therefore have a range of positive educational, behavioral, and psycho-social outcomes (11, 12).

The socio-cultural, linguistic, and educational circumstances of Indigenous youth in remote communities of the NT and the profile of risks and challenges they face, underscore the need to establish the feasibility of school-based prevention. These students are at very high risk but at the same time are least likely to have access to appropriate evidence-informed interventions and supports. Moreover, despite recent interest in measurement of resilience and social-emotional well-being of Indigenous adolescents, there is limited evidence for the appropriateness and reliability of these instruments in very remote populations (13). To establish whether a culturally adapted universal curriculum delivered by school teaching staff can contribute to improved social-emotional learning and reduced risk it is necessary to determine what methods and instruments are suitable for the evaluation of such interventions.

## The Current Study

At commencement of the phase of research reported in this study, Skills for Life (SFL) had been developed through a 2-year process of engagement of the researchers with community leaders, youth workers, and educators as well as parents in a remote community school in the West Arnhem Region of the NT, 500 km from the provincial capital of Darwin. This was in effect a form of participatory action research (PAR) with continuing collaboration, evaluation, and feedback spanning curriculum development and teaching with ongoing monitoring of risks by the teachers and the school's resident psychologist in consultation with the research team.

The community is complex, with a population of just above 2,300 persons and at least seven distinct Aboriginal languages spoken by residents. It acts as a hub for 30 or more outstations on surrounding traditional lands, with considerable movement of the population between them for reasons of traditional ceremony and seasonal subsistence living preferences. Overall student attendance has ranged from 55 to 43%, in the years from 2014 to 2019, with from 13 to 5% of students attending school 90% or more of the time (3), and appears to have declined despite government-funded proactive strategies aiming to maximize student participation. At the time of this project, research officers had resided in the community over months and worked with local assistants at school and at the community youth center, using a range of strategies to engage the parents of young people in activities and information sessions.

Content and approach of the SFL curriculum had been developed from 2012 to 2013 through workshops and consultations with knowledgeable Indigenous elders, Indigenous school staff, and classroom teachers (14). Intended as a resource for school-based suicide prevention for students in years 7, 8, and 9, SFL consisted of 12 weekly lessons taught by school staff who received training and were provided with lesson plans, resources, and ongoing telephone support. The locally based school psychologist was experienced in delivery of both student counseling and public health interventions in remote community settings and was extensively consulted on methods of the program and about children's at-risk behavior.

This study describes a preliminary trial of methods of data collection using a series of psychometric measures to assess their feasibility and suitability for the evaluation of the SFL program. After collaborating in its development, the school had committed to teach the curriculum and to its longer-term evaluation. It was, therefore, necessary to incorporate a preliminary trial of evaluation methods and measures in the context of ongoing program delivery in order to establish their appropriateness and feasibility for the continuing project, including eventual replication in other remote schools. At this stage of development of the program, the participation of other schools had not been secured, and for reasons of cost and the burden on school and staff resources, it was not possible to separately pilot instruments with a comparison sample in other schools.

The specific aims of this preliminary pilot project were as follows:

- to test methods of engagement and data-collection in the context of continuing delivery of the program,
- to assess the appropriateness, reliability, and validity of specific outcome measures when administered to students withdrawn from class,
- to undertake exploratory modeling of pre- and post-program changes for the pilot program using the trial measures, and
- to monitor possible adverse effects of participation in the program.

## Methods

### The Program

The 12-lesson SFL program was taught across two school terms (terms 2 and 3) in 2014 and 2015 to allow for periods of data-gathering at pre- and post-intervention. The lessons were team-taught by the students' normal classroom teachers and a member of the research team who was an experienced, registered educator with a background in literacy teaching. Lessons were taught in Health and Physical Education (HPE) periods, once a week for 90 min resulting in a total possible exposure of 18 SFL hours (12 weeks × 90 min).

In 2014, SFL was taught in two single sex middle years classrooms including children from years 7 to 9 with relatively high achievement and attendance. In 2015, the evaluation spanned three classes at the same year levels, one intervention class and two classes as a comparison group. The intervention class was co-educational and streamed by the school as a “high attendance, high engagement” class, while two comparison classes consisted of students with lower engagement and attendance.

### Data-Gathering

The research team developed a standardized protocol for data-gathering. Instruments were administered in standard English with a small number of items modified with the advice of local community assistants. A standard set of alternative phrases was developed to be used if students signaled that they did not understand an item. Research assistants were trained to administer questionnaires to young people of differing literacy levels. During the 2 weeks pre- and post-program, students left the classroom in ones and twos with a researcher and sat in a quiet room set aside for the survey. Some students volunteered to read and complete the questionnaire themselves. For most students, the items were read out in a neutral voice by the researcher, with the student then marking his or her response on the scale. A protocol for response to indications of risk or distress among students during teaching and data-gathering was agreed by the psychologist and teachers in consultation with the research team.

### Ethics and Parental Consent

Ethics approval was granted by the HREC of the NT Department of Health and the Menzies School of Health Research 2013–2120 in March 2014 and approval to conduct research was given by the NT Department of Education. After the 1st year of the pilot, changes in the procedure for consent were approved by the HREC.

In 2014, following practices adopted for the development phase of the project, all parents were contacted by the researchers to provide information about the project and seek written consent for data-gathering and analysis. However, it proved to be practically impossible for the researchers to obtain consents prior to commencement of data-gathering and lesson delivery due to difficulty contacting parents, who were highly mobile and frequently unavailable for extended periods, often absent visiting outstation communities, for example. Despite repeated follow-up, in 2014 parents of 26 students could not be contacted. These students thus participated in the program but were excluded from the current analysis.

After the 1st year of the pilot, the College leaders and the NT Department of Education were consulted about procedures for consent. It was accepted that the program was a low risk program taught by school staff and that evaluation instruments were consistent with the school's educational aims in adopting the curriculum. It therefore met the requirements for “standing order of consent” with provision for parents to “opt out” of participation in the study and for consent to be otherwise assumed (15). The school agreed to provide information to parents about SFL in advance of teaching, to explain the evaluation to them, and their ability to request further information and/or to formally “opt out.” Although there were a small number of requests for further information, no parents opted out of the evaluation in 2015.

### Participants

As a result of differences in inclusion criteria and in data available in each of the 2 years, for purposes of analysis, the 2 years of teaching and data collection are treated as separate studies, Study 1 in 2014, and Study 2, 2015.

The Study 1 sample comprised 15 students in grades 7–10 with both pre- and post-intervention measurements and parental consent (Table 1). This is after an attrition rate of 40% with 10 lost to follow-up from the original 25 participants with pre-intervention measurements (excluding those students without parental consent). This attrition reflects the low average school attendance rate at the participating school of 55% in 2014, (3) and classroom attendance of the study sample (Mean (*M*) = 68.5%, Standard deviation (*SD*) = 21.7, ranging from 27.1 to 97.9%).

**Table 1:** Sample characteristics for two phases.

	**Phase**		
	**Study 1**	**Study 2**	**Studies 1 and 2**
Number of participants	15	48	8
Number of intervention classes: comparison classes	2:0	1:2	
Percent of sample in the intervention	100	30.6	
Attrition rate (%)	40.0	33.3	
Gender: females (%)	33.3	35.4	
Age: year mean (SD)	13.6 (0.74)	13.4 (0.98)	
Range	12–15	11~15	

In Study 2, the final sample included 48 students out of a total of 72 students. The attrition rate was 33.3% (*N* = 24) reflecting the students' low general school attendance rate. The intervention group data were from 17 students whereas 31 students provided their data in the comparison group. Eight students (35.3%) participated in the intervention program for 2 years, so any possible dosage effect was analyzed supplementarily.

### Measures

In the two pilot studies, measures administered to participating students were the Strengths and Difficulties Questionnaire (SDQ) student self-report version (17) as primary outcome measure; the Kessler 6 (K6), (16), and a purpose designed scale developed to assess the relevance of the construct of connectedness to student resilience, referred to here as the Connected Self Scale (CSS).

The Strengths and Difficulties Questionnaire (SDQ) consists of 25 items in five subscales and is used both as a clinical assessment and screening tool and as an evaluation measure (17, 18). Parent and teacher versions have been used with Australian Indigenous populations (19, 20). However, there are no published data on use of the self-report version with remote Indigenous youth.

The K6 is a six-item measure of general psychological distress. The K6 has been validated with Australian and international samples as a screening scale for mental health and psychological well-being (21). It is widely used with reportedly high reliability across different populations and socio-cultural contexts and found suitable for use with adolescents of both genders and different ages, with support for a one-factor model of psychological distress and a two-factor model of depression and anxiety (22–24). For scoring used in this study, K6 scores >10 are taken to indicate moderate psychological distress, while scores >18 indicate severe distress and high risk of depression or anxiety disorders (25).

The Connected Self Scale (CSS) was developed by the researchers after a review of measures of youth resilience used in school settings, including the Middle Years Development Instrument, which includes a domain of connectedness (26). It was intended as an exploratory trial of the utility of dimensions of connectedness as indicators of external supports for resilience. The CSS had 14 items in 4 subscales, Self-Concept; Home Support, School Connectedness, and Community Connectedness. It included items such as: “In general, I like being the way I am”; “At home, there is a parent or other adult who really cares about me”; “At my school, there is a teacher or other adult who always wants me to do my best” and “In my community, there is an adult who listens to me when I have something to say.” The response scale consisted of four points ranging from “Not at all True” (2) to “Very much True” (5).

Descriptive statistics (Table 2) show that the reliability of 11 scales (five subscales and Total Difficulties scale of SDQ, four subscales of CSS, and the K6 scale) ranged from −0.11 (SDQ Total Difficulties pre-, Study 1) to 0.79 (SDQ Emotional symptoms post, Study 1). The K6 was relatively reliable (α = 0.60 −0.66) compared to other measures for both samples. Because the Home support scale consisted of five items compared to three items each for the other subscales, the proportional advantages in Cronbach's alpha reliability to the number of items almost certainly impacted results for the CSS subscales. The conventional standard for adequate reliability is a Cronbach's alpha larger than 0.70. However, moderate reliabilities for adolescent samples are not uncommon (27), so that a lenient cut off for this pilot study was applied.

**Table 2:** Cronbach's Alpha (α) for all scales: scales with asterisks^*^ selected for analysis and values shown in bold.

**Measure**	**Subscale**	**α** **(2014)**		**α** **(2015)**	
		**Pre**	**Post**	**Pre**	**Post**
Strengths and Difficulties Questionnaire	Conduct	0.44	0.52	0.18	0.09
	Emotional	−0.44	0.79	0.64	0.32
	Hyperactivity	0.30	0.61	−0.45	−0.45
	Prosocial^*^	**0.60**	**0.79**	0.47	0.49
	Peer	−0.16	0.05	−0.17	−0.09
	Total Difficulties	0.19	0.69	0.53	0.23
Kessler 6^**^		**0.61**	**0.60**	**0.61**	**0.66**
Connected Self Scale	Self^*^	**0.64**	**0.54**	0.33	0.59
	Home^**^	**0.73**	**0.50**	**0.53**	**0.62**
	School	0.34	0.38	0.37	0.39
	Community	0.25	0.53	0.45	0.65

Conduct, conduct problems scale; Emotional, emotional symptoms scale; Prosocial, prosocial behavior scale; Peer: peer problems scale;

* Variables analyzed in Study 1;

** *Variables analyzed in both Study 1 and 2 based on scale reliability*.

We selected variables for analysis only when the α was close to or above 0.60 for *both* pre- and post- measurements (Table 2). As a result, four measures, (the K6, the prosocial behavior scale of SDQ, the Self-concept and Home connectedness scales of the CSS) were analyzed with the 2014 data set. For the data from the 2015 cohort, only the K6 and the home connectedness scale of the CSS were analyzed.

### Analysis Overview

First, descriptive statistics and attrition analysis for Study 1 and Study 2 are presented. To test for associations between exposure to the intervention and pre- and post-program changes, the Study 1 data were analyzed with repeated measures ANOVA using SPSS. For Study 2 data, mixed model ANOVA was conducted with the two-time point repeated measures and a between-subject factor with the two levels of intervention and comparison groups. A dosage variable was also included. For Study 1, attendance at intervention sessions was categorized into low and high attendance groups. This dosage between-subject group variable was used in the subsequent mixed ANOVA with the repeated pre- and post- measures.

For Study 2, attrition and attendance data were not available. However, yearly dosage was available for analysis. Students within the intervention group who participated in the intervention program for 2 years were compared with those who participated for only 1 year. Further, three dosage groups including the comparison group with no intervention were also compared. In addition to the mixed ANOVA, supplementary regression analysis was also conducted to cope with the unbalanced cell sizes among the three dosage groups.

Further supplementary analyses were conducted to increase the power for the small samples. No further significant results were found from these analyses.

## Results

### Study 1 Cohort

#### Descriptive Statistics

Data screening showed that the SDQ prosocial behavior variable measured at post-intervention (Kolmogorov-Smirnov test, *p* = 0.009) and CSS self-concept (Kolmogorov-Smirnov test, *p* = 0.047) at the baseline were not normally distributed whereas the K6 and the home connectedness variables satisfied the normality assumption test. Accordingly, the bootstrapping method was used to estimate the confidence intervals in the main analysis for the two variables that violated the normality assumption. Descriptive statistics and correlations of the study variables are presented in Table 3.

**Table 3:** Correlations and descriptive statistics of the study variables, Study 1 (*n* = 15).

	**Pre-K6**	**Post-K6**	**Pre-SDQ Prosocial**	**Post-SDQ Prosocial**	**Pre-CSS Self-concept**	**Post-CSS Self-concept**	**Pre-CSS Home-support**	**Post-CSS Home-support**
Pre-K6	–							
Post-K6	0.36	–						
Pre-SDQ Prosocial	−0.20	−0.13	–					
Post-SDQ Prosocial	−0.01	0.10	0.55^*^	–				
Pre-CSS Self-con	−0.06	−0.47	0.69^**^	0.30	–			
Post-CSS Self-con	−0.20	−0.39	0.79^**^	0.62^*^	0.83^**^	–		
Pre-CSS Home	−0.14	−0.13	0.71^**^	0.33	0.60^*^	0.57^*^	–	
Post-CSS Home	0.13	−0.21	0.51	0.58^*^	0.60^*^	0.72^**^	0.65^**^	–
Mean	16.13	16.40	8.27	8.60	3.38	3.20	2.85	2.96
SD	4.24	4.44	1.67	1.77	0.68	0.73	0.73	0.62

DQ, Strengths, and difficulties questionnaire, prosocial subscale. Pre, pre-intervention. Post, Post-intervention. CSS, Connected Self Home support, and Self-concept subscales. Scale. SD, Standard Deviation.

* p < 0.05.

** *p < 0.01*.

#### Attrition Analysis

The four well-being measures were compared between the students who dropped out from the post-intervention measurement (*n* = 10) and the students who participated in both pre- and post- measurements (*n* = 15). No significance differences were found for K6 [*M (SD)*_*fullparticipation*_ = 16.13 (4.24), *M (SD)*_*dropouts*_ = 17.10 (2.23), *F*_(1, 23)_ = 0.44, *p* = 0.516]; SDQ prosocial [*M (SD)*_*fullparticipation*_ = 8.17 (2.68), *M (SD)*_*dropouts*_ = 9.00 (1.05), *F*_(1, 23)_ = 1.52, *p* = 0.231]; CSS self-concept [*M (SD)*_*fullparticipation*_ = 3.38 (0.68), *M (SD)*_*dropouts*_ = 3.57 (0.39), *F*_(1, 23)_ = 0.64, *p* = 0.434]; CSS home connectedness [*M (SD)*_*fullparticipation*_ = 2.85 (0.73), *M (SD)*_*dropouts*_ = 3.05 (0.51), *F*_(1, 23)_ = 0.58, *p* = 0.454]. The absence of attrition effects supported the generalizability of the main analysis results.

#### SDQ Prosocial Behavior

**Figure 1:**
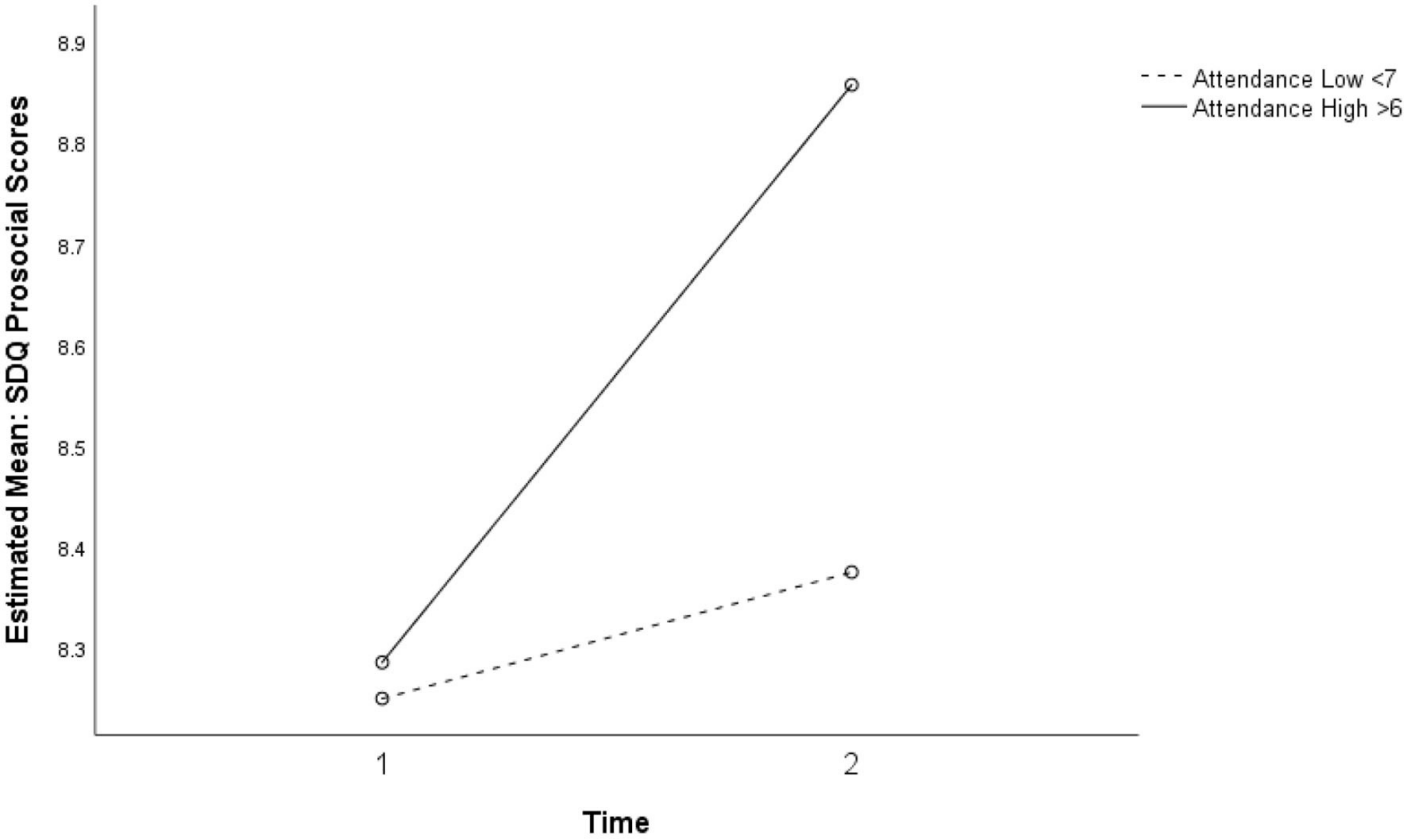
Non-significant interaction for prosocial behavior between intervention, attendance, and time (*p* = 0.616).

There was no significant association between the intervention and change in the SDQ prosocial behavior subscale. However, as shown in Figure 1, a trend of interaction was observed between intervention attendance and time, without statistical significance. The participants with higher intervention attendance appeared more likely to report increased prosocial behavior after the intervention compared to the participants with low attendance. There were increased scores in prosocial behavior for 40% of the participants (*n* = 8) after the intervention, with the change score ranging from −2 to 3 (*M* = 0.33, *SD* = 0.42).

#### Psychological Distress (K6)

No significant association with the intervention was observed for psychological distress. However, at the individual level, over half of the students (*n* = 8, 53.3%) showed decreased K6 scores after the intervention with the sample mean change score of −0.27 (SD = 1.27) ranging from 14 to 6.

#### CSS Self Concept

There was no significant association between intervention and change in participants' self-concept. More than half of the participants (*n* = 8, 53.3%) showed the same scores of self-concept after the intervention suggesting stability in self-concept.

#### CSS Home Support

**Figure 2:**
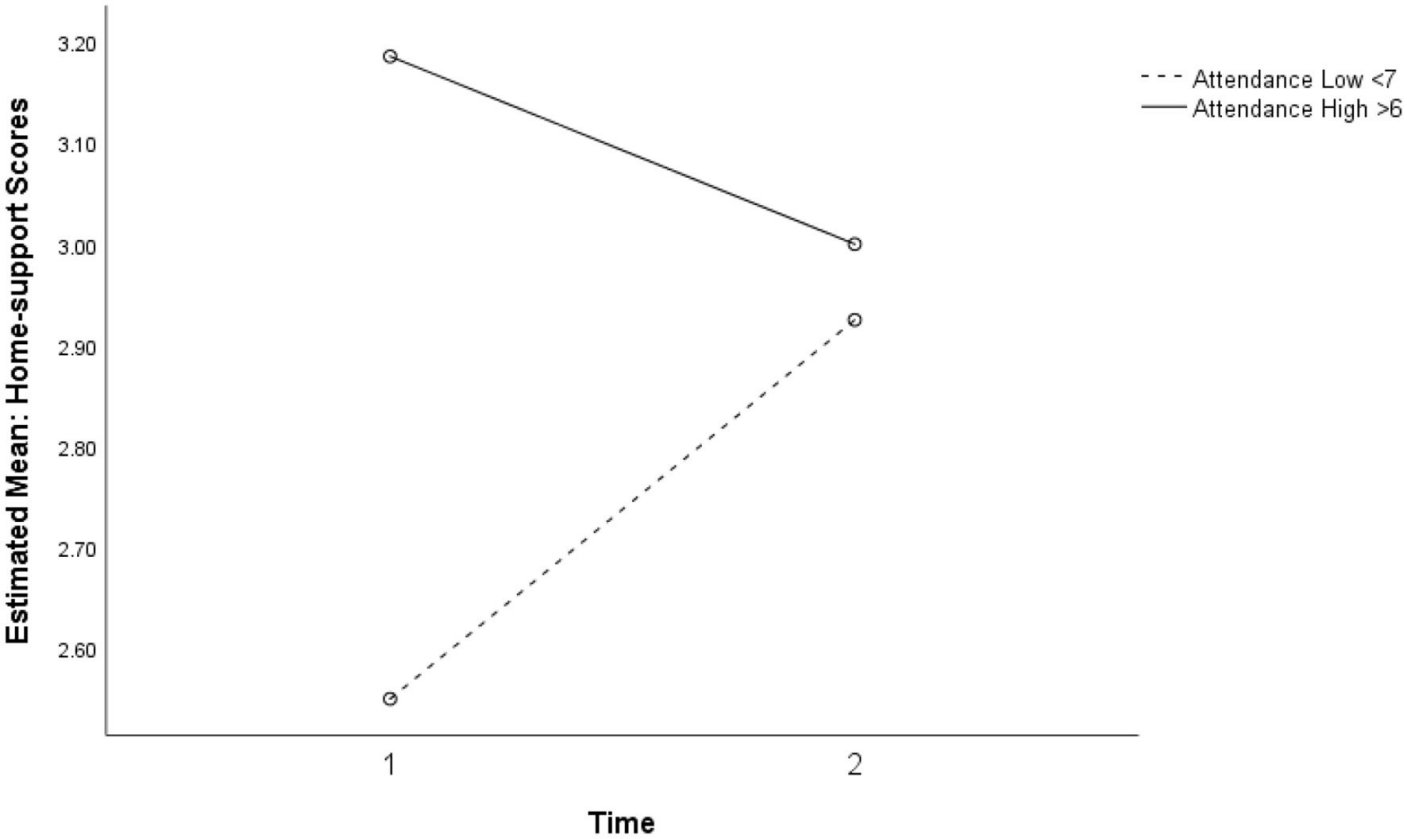
Marginally significant interaction between intervention attendance and time for perceived home support (*p* = 0.056).

Two-way repeated ANOVA showed a near-significant interaction between time and intervention attendance for perceived home support, *F*_(1, 13)_ = 4.39, *p* = 0.056, η_p_^2^ = 0.252, power (1–β) = 0.49. As presented in Figure 2, participants who attended the intervention sessions for <7 weeks showed improved perception of home support at Time 2, while participants who attended the intervention sessions for more than 6 weeks reported higher levels of home support at both time points.

### Study 2 Cohort

#### Descriptive Statistics

Data screening showed that the CSS home connectedness variable measured at post-intervention was not normally distributed (Kolmogorov-Smirnov test, *p* = 0.018) whereas the K6 variable satisfied the normality test. The bootstrapping method was used to estimate the confidence intervals in the main analysis to deal with the violation of the normality assumption. Descriptive statistics and correlations of the study variables are presented in Table 4.

**Table 4:** Correlations, Study 2 (*n* = 48).

	**Pre-K6**	**Post-K6**	**Pre-CSS Home-support**	**Post-CSS Home-support**
Pre-K6	–			
Post-K6	0.35^*^	–		
Pre-CSS Home-support	−0.01	−0.06	–	
Post-CSS Home-support	−0.09	0.11	0.25	–
M	16.73	17.29	2.57	2.91
SD	4.67	4.78	0.66	0.66

Pre, pre-intervention. Post, post-intervention. CSS, connected self scale.

* *p < 0.05*.

#### Attrition Analysis

The two well-being measures were only examined if the students who dropped out from the post-intervention measurement (*n* = 24) had significantly different levels from the students who participated in both pre- and post- measurements (*n* = 48). The two groups were not significantly different for K6 [*M (SD)*_*fullparticipation*_ = 16.73 (4.67), *M (SD)*_*dropouts*_ = 17.04 (4.11), *F*_(1, 70)_ = 0.08, *p* = 0.782] and CSS home connectedness [*M (SD)*_*fullparticipation*_ = 2.57 (0.66), *M (SD)*_*dropouts*_ = 2.62 (0.51), *F*_(1, 23)_ = 0.11, *p* = 0.747]. Because no attrition effects were found the main analysis results could be generalized.

#### K6 Psychological Distress, Study 2

Two-way repeated ANOVA did not show any significant association of intervention with K6 scores between the intervention group (*n* = 17) and comparison group (*n* = 31). Individual level change scores were not significantly different between the two groups. Although the difference was not significant, χ^2^ (1, *n* = 48) = 0.117, *p* = 0.483 (Fisher's Exact Test, 1-sided), a slightly greater proportion of participants (*n* = 8, 47.1%) in the intervention group showed decreased K6 scores than in the comparison group (*n* = 13, 41.9%). Lacking attendance data in 2015, no further analysis was conducted.

**Figure 3:**
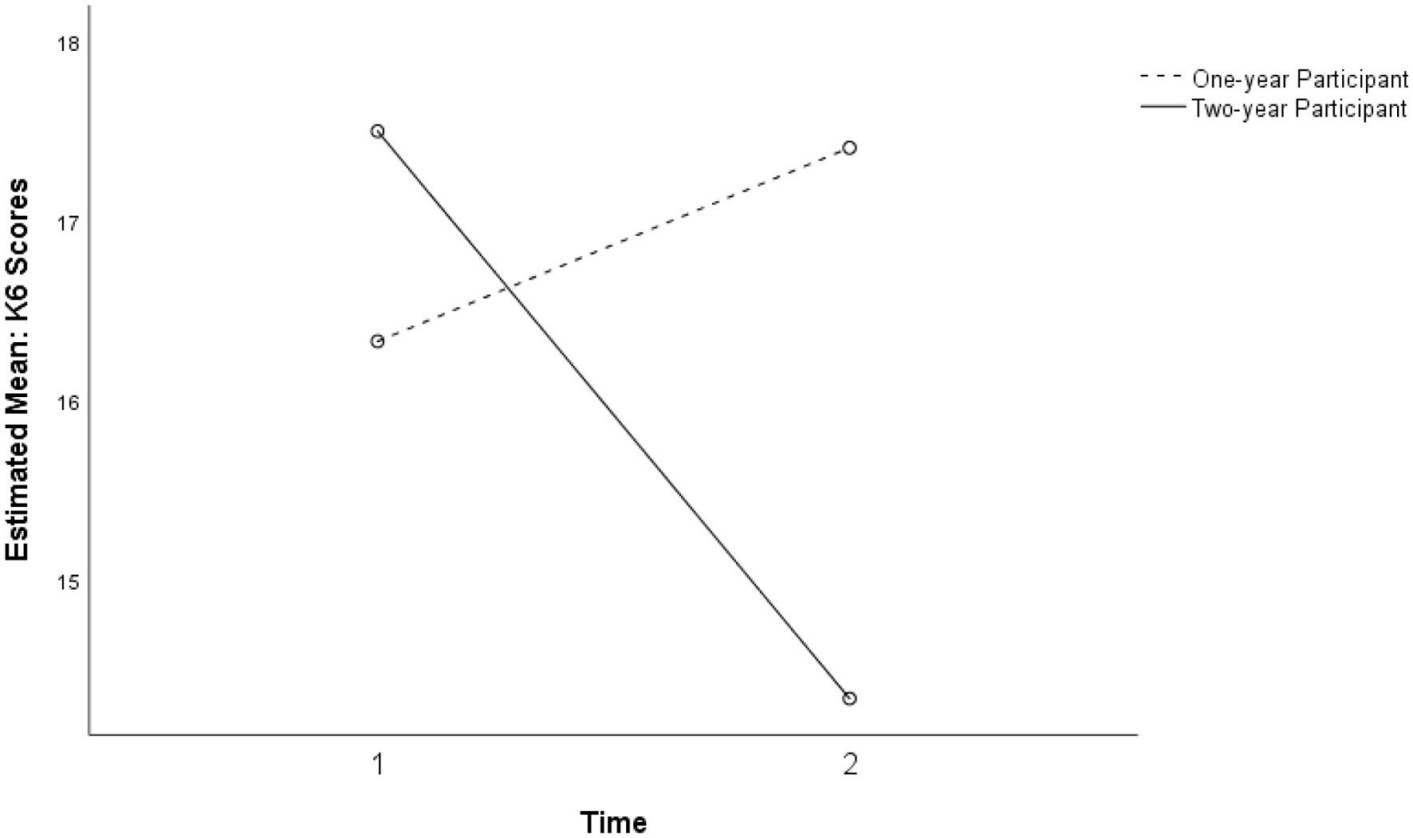
Near-significant interaction between time and intervention dosage for K6 (*p* = 0.082).

**Figure 4:**
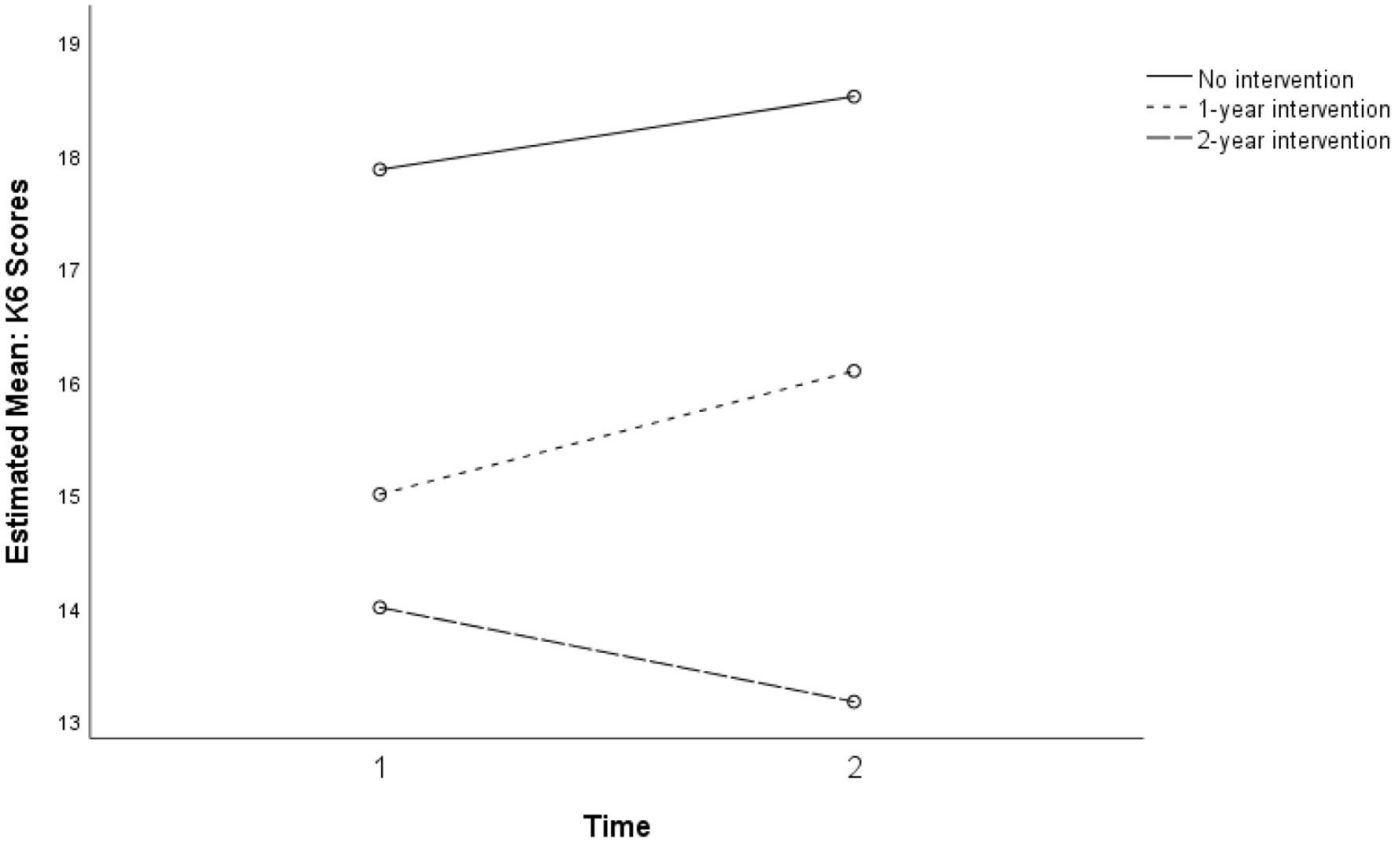
Interaction between time and intervention dosage for K6 for treatment and comparison groups (*p* = 0.067).

A three-way repeated ANOVA showed an interaction between time and dosage in explaining the variance in K6 scores over two phases, *F*_(1, 44)_ = 3.16, *p* = 0.067, η_p_^2^ = 0.130, power (1–β) = 0.41, that was just short of significant. Figures 3, 4 show that students who participated in the intervention program for 2 years (*n* = 8) showed a decrease in K6 scores whereas others showed a slight increase.

**Figure 5:**
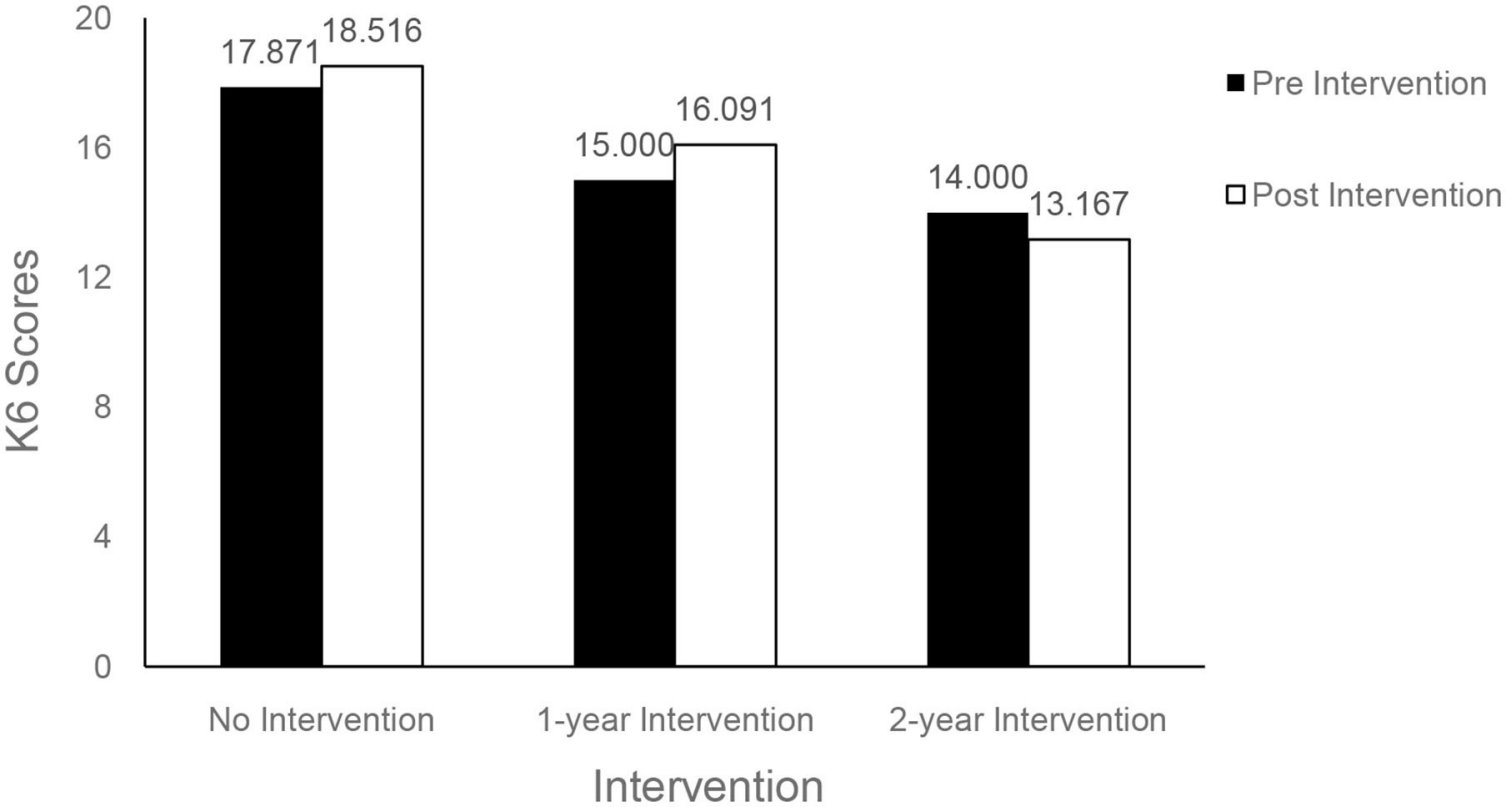
Mean K6 scores, pre-, and post-intervention.

For the K6 measure of psychological distress, a significant association with dosage was observed (see Figure 5 below). Compared to the comparison group (*n* = 31), students who participated in the intervention program for 2 years (*n* = 8) were more likely to have decreased K6 scores (*b* = −4.36, *SE* = 2.04, β = −0.31, *p* = 0.038) at the post-intervention measurement when the pre-intervention score was controlled as a covariate. The model explained 20.9% of the variance in K6 scores at post-intervention with marginal significance (*p* = 0.10).

#### Home Support, Study 2

**Figure 6:**
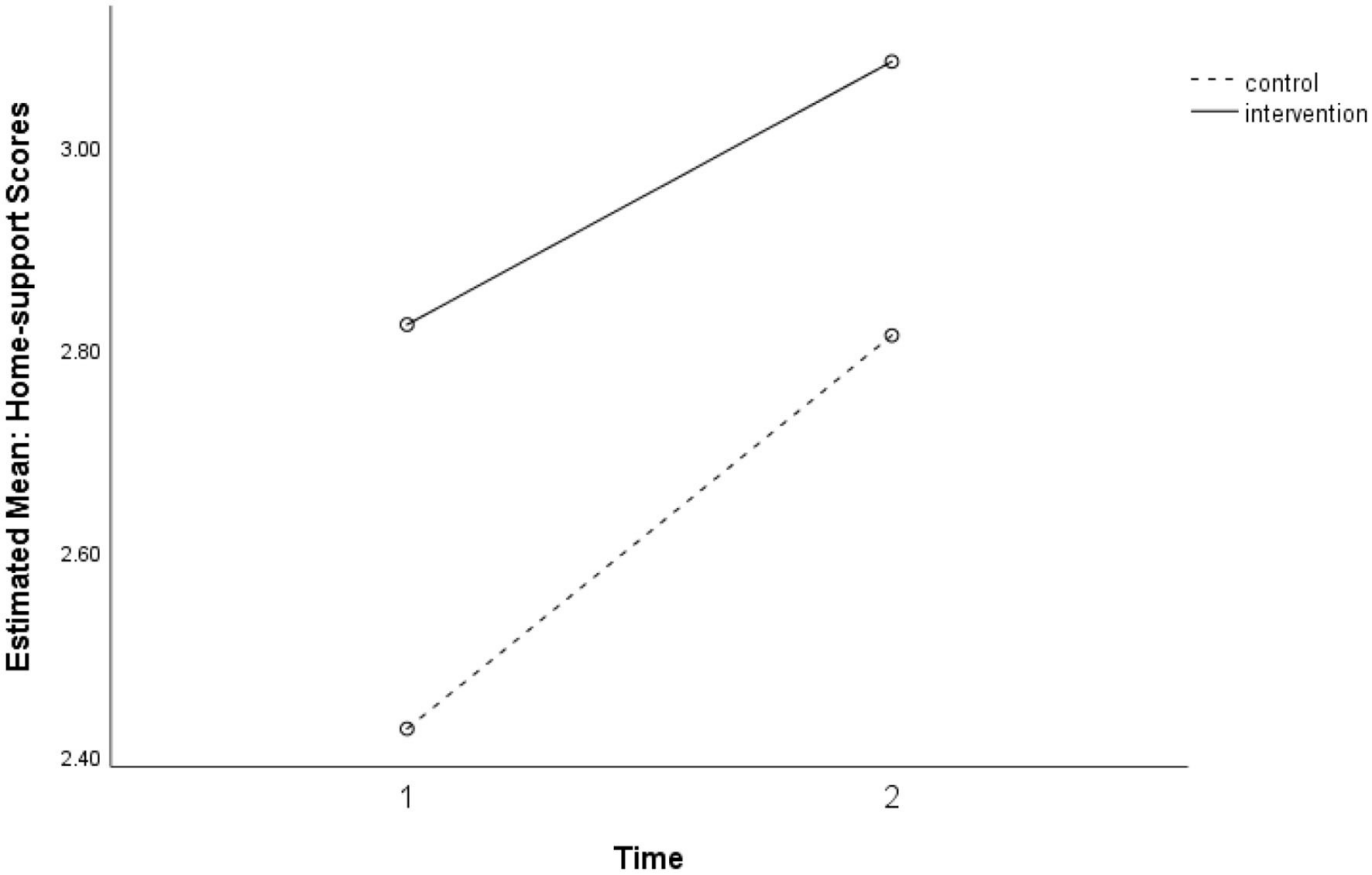
Time and perceived home support for intervention and comparison groups (*p* = 0.012).

Two-way repeated ANOVA showed a significant association between time and perceived home support, *F*_(1, 46)_ = 6.86, *p* = 0.012, η_p_^2^ = 0.130, power (1–β) = 0.73. As presented in Figure 6, participants from both intervention and comparison groups showed higher perceptions of home support at Time 2 (*n* = 48). No significant difference between the two groups was observed. For home connectedness, no significant association was found from the regression analysis.

**Figure 7:**
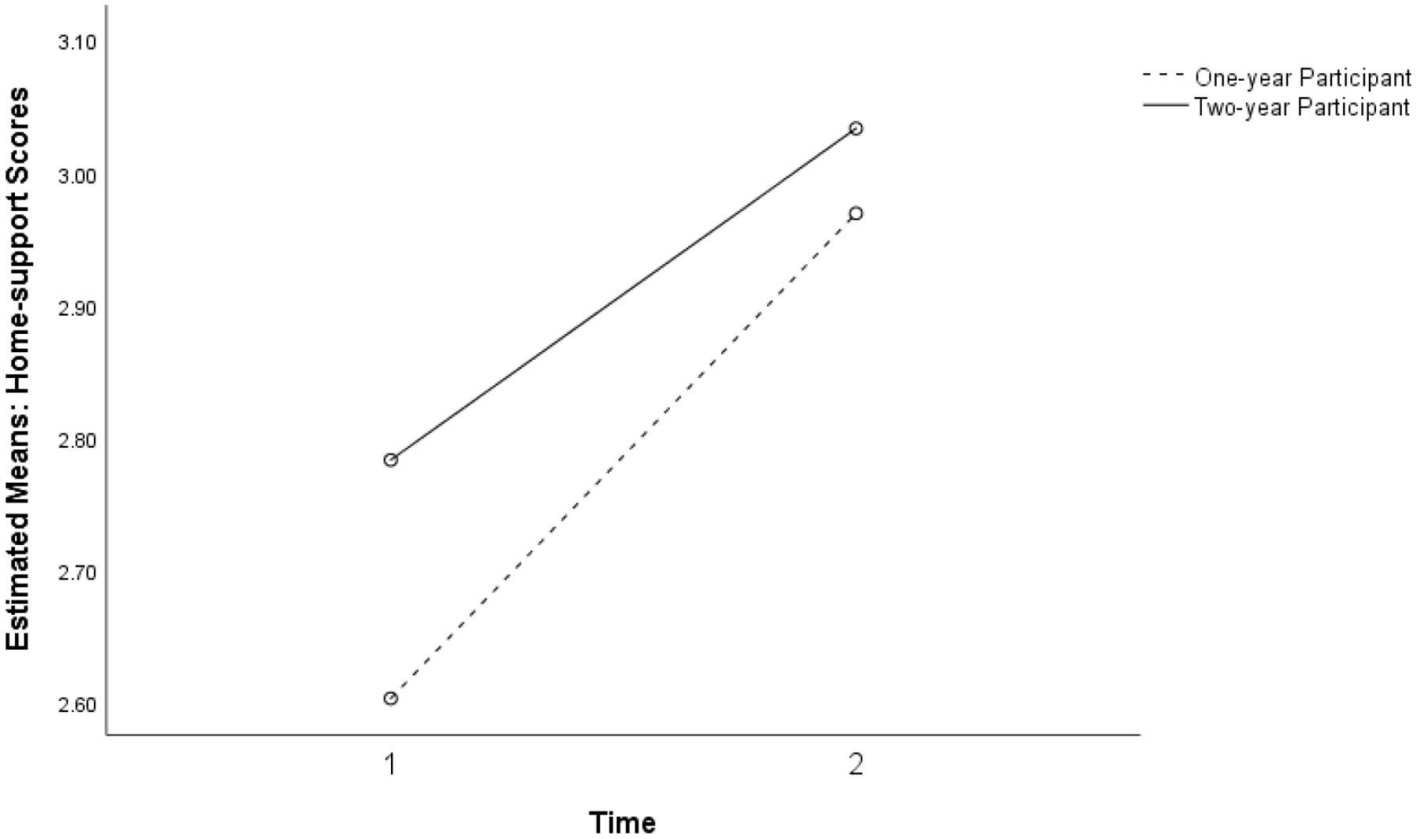
Non-significant interaction between the intervention dosage and intervention for perceived home support in study 2 for the 1-year participant and two-year participant groups (*p* = 0.012).

Although the intervention dosage over 2 years did not show significant main effects or interaction effects with the Study 2 intervention group, students who participated in the previous year's intervention showed an increase in positive perceptions of home support at Time 2 (Figure 7).

**Figure 8:**
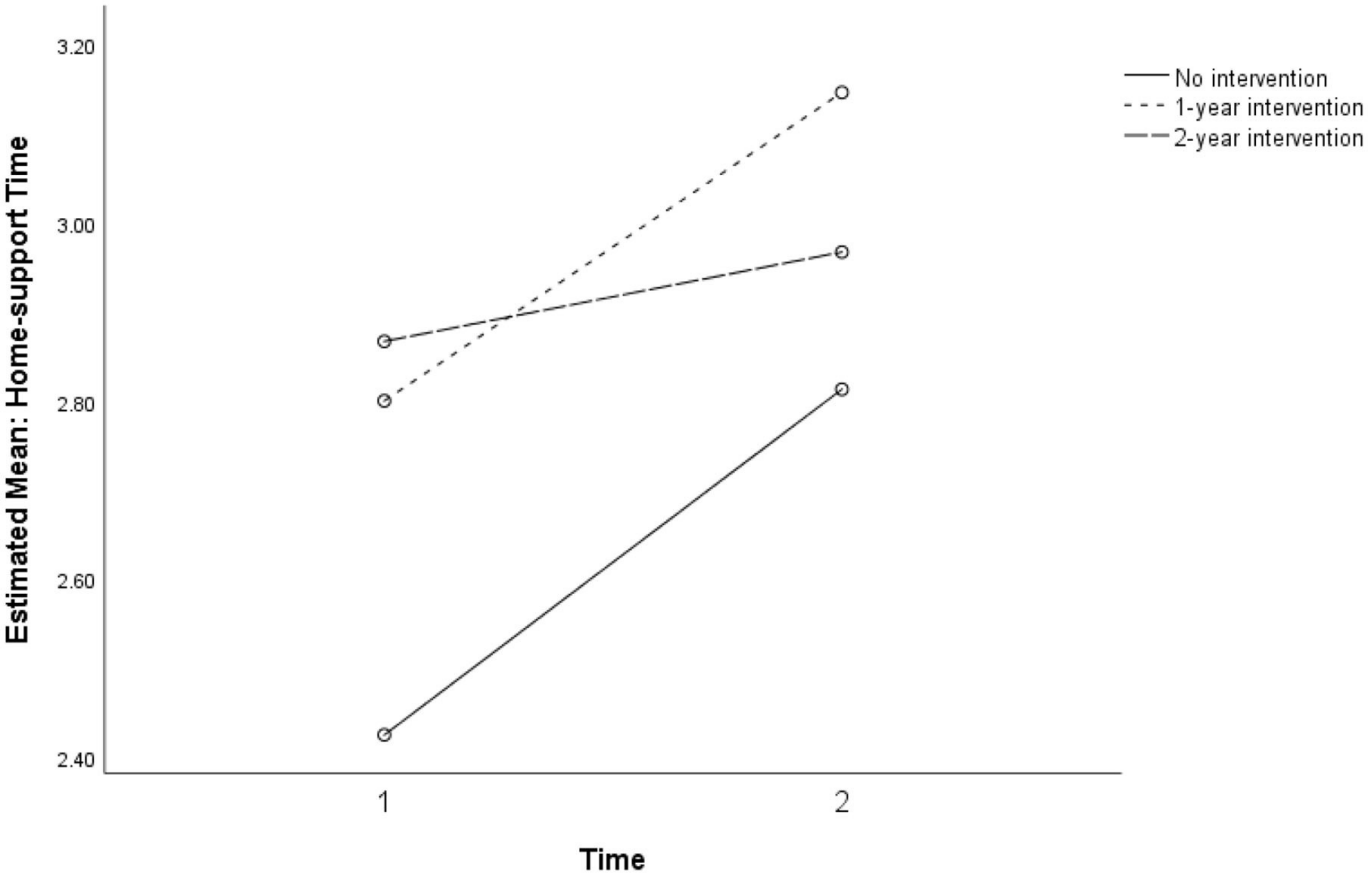
Non-significant interaction for perceived home support between intervention dosage in three groups: no intervention, 1-year, and 2-year intervention dosages (*p* = 0.738).

Figure 8 shows the mixed ANOVA results with a non-significant interaction between time and dosage for the three levels of no intervention, 1-year intervention, and 2-year intervention. Because the cell sizes of the three dosage groups were unbalanced, regression analysis using dummy codes of the group membership was conducted.

## Discussion

This study presents the results of a feasibility pilot of a curriculum-based intervention, Skills for Life, delivered to Australian Indigenous students in years 7–9 middle school classes over 2 years at a remote community school. The aims of this phase of the pilot program were to assess the feasibility and appropriateness of youth self-report measures of student difficulties, strengths, psychological distress, and connectedness when administered at interview and to identify the direction of changes pre- and post-intervention to inform decisions about the choice of measures for a planned extension of the pilot program and its evaluation.

### Scale Performance

Three measures consisting of 11 scales were administered to the students pre- and post-program. Only four scales achieved reliability at either appropriate or marginally considerable levels in either the Study 1 or Study 2 samples: the K6, the Prosocial behavior scale of the SDQ, and the Self-concept and Home support subscales of the CSS as developed by the research team.

Normative data for the youth self-report version of the SDQ for a sample of Australian students in the general population suggest moderate indices of internal consistency for four of five subscales, and an acceptable Cronbach's alpha for total difficulties (18). However, although parent and teacher versions of the SDQ are widely used to assess adolescent well-being, reliability indices for Indigenous samples have not been reported in recent studies including a systematic review of 11 surveys (28, 29). There are few published reports of the properties of the youth self-report version of the SDQ internationally (18, 27), and none for Australian Indigenous youth in either intervention studies or surveys.

International studies of the SDQ self-report have found moderate indices of internal consistency for subscales, and questions about the factorial structure (27, 30). A study concluded that evidence for the validity of the factorial structure of the SDQ with many minority cultural groups in the U.S. is poor (31). It surveyed English-speaking *Latinx* youth and tested the existing subscales with confirmatory factor analysis and conducted exploratory factor analysis to identify any further factors with this sample. After testing 5-, 3-, and 2-factor solutions, a two-factor model of difficulties and prosocial behaviors performed best. According to the authors, a significant correlation between the two factors suggested that low levels of distress and difficulty do not imply the presence of strengths.

For the two samples reported here, there was little consistency between pre- and post-program Cronbach's alpha values both for the five subscales and for the total difficulties scale, with only the prosocial scale showing acceptable values at pre- and post-program with the 15 cases of Study 1 (Table 2). There was even greater inconsistency in the larger Study 2 sample. Together with the lack of published analysis of the properties of the youth self-report version among comparable Indigenous populations the findings of the preliminary analysis were considered sufficient to justify discontinuing the use of the SDQ-SR for this project.

Although the properties of the K6 as a measure of psychological distress have been described in a general population sample of Australian adolescents, psychometric properties of the K6 have not been reported for Indigenous samples in a review of 32 studies (32) and other research on Indigenous mental health (33, 34).

Of the scales used for the current study, only the K6 showed moderate to acceptable Cronbach's alpha values across both measurements for both study samples. Preliminary findings confirm the utility of the K6 as a secondary outcome measure for the evaluation of SFL and justify further study of the program's short- and long-term impacts on high levels of distress and to further investigate correlates of distress among Indigenous students.

Of the four subscales of the Connected Self Scale developed by the research team, the Home support scale showed adequate internal consistency across both Study 1 and 2 samples, while the Self-concept scale was reliable only in Study 1. These findings suggest that further development of the connectedness scales or adoption of a similar existing scale would be justified for this student population. Future evaluation of the program should measure the significance of Indigenous students' perception of external supports across home, school, and community and their contribution to students' capacity to cope with high levels of psychological distress.

### Characteristics of the Study Population

The analysis has identified some important characteristics of the sample of participants. K6 scores pointed to very high levels of distress in this population with high mean scores for all classes compared with general Australian samples (21). The prevalence of scores >18, indicative of serious mental illness, ranged from 28.6 to 38% across all samples pre- and post. Significant differences between mean levels of psychological distress in the intervention and comparison classes in Study 2 (Figure 6) are likely to suggest actual differences in levels of distress experienced within groups in the sample. Both low attenders in Study 1, and non-intervention classes in Study 2 reported lower levels of Home support compared with high attenders and the intervention classes, respectively (Figures 2, 7). The non-intervention classes consisted of students who were classed by the school as infrequent attenders with low academic engagement and it is not implausible that this group would experience higher levels of distress and lower home support than others with high attendance and academic achievement.

A large-scale study in the USA used latent class analysis to identify multiple subtypes of serious mental illness measured by the K6 that were predicted by age, family structure, substance misuse, antisocial behavior, role impairment, and peer victimization (35). An extended study of student well-being and psychological distress in Australian remote communities should investigate different components of mental distress and predictors among the student population. These are areas for development of the evaluation strategy for preventive programs like SFL.

### Change Scores: Students' Response to SFL

Analysis of change over the time of intervention for both Study 1 and Study 2 samples produced limited positive results, summarised in Tables 5–7. However, these did highlight questions for further investigation. Students who attended the SFL program for 2 years showed a near-significant decrease in level of psychological distress (K6) that was significantly lower when compared to other students with 1-year participation or no intervention in the comparison groups.

**Table 5:** Model summary.

					**Change Statistics**				
**Model**	***R***	***R*^**2**^**	**Adj. *R*^**2**^**	**SE**	***R*^**2**^ change**	***F* change**	***df*1**	***df*2**	***p F* change**
1	0.349^a^	0.122	0.103	4.523	0.122	6.390	1	46	0.015
2	0.457^b^	0.209	0.155	4.390	0.087	2.417	2	44	0.101

a Predictors: (Constant), k6 Pre-intervention score.

b *Predictors: (Constant), k6 pre-intervention score; Dosage 2, 2-year intervention effect, Dosage 1, 2-year intervention effect*.

**Table 6:** Coefficients.

		***B***	***SE B***	***B***	***T***	***p***	**Zero-order**	**Partial**	**Part**
1	(Constant)	11.313	2.454		4.610	0.000			
	Pre_k6tot	0.357	0.141	0.349	2.528	0.015	0.349	0.349	0.349
2	(Constant)	13.936	2.724		5.116	0.000			
	Pre_k6tot	0.256	0.146	0.250	1.757	0.086	0.349	0.256	0.236
	DosageDum1 2-year intervention effect	−4.357	2.038	−0.305	−2.138	0.038	−0.330	−0.307	−0.287
	DosageDum2 1-year intervention effect	−1.689	1.597	−0.150	−1.058	0.296	−0.139	−0.158	−0.142

*Pre, Pre-intervention. Post, Post-intervention. Dosage 1, dummy variable for 2-year intervention effect. Dosage 2, Dummy variable for 1-year intervention effect*.

**Table 7:** Results summary.

	**Outcomes**	**Effects**	**Note**
Study 1	K6	Not significant	50% participants: decreased scores
	SDQ ProSocial	Not significant	A trend toward increased scores for participants with high intervention attendance
	CSS Self-Concept	Not significant	50% participants: no change
	CSS Home Support	Marginally significant interaction effect	High attendance group retained higher perceptions of home support
			Low attendance group showed increased home support perceptions
Study 2	K6	Marginally significant interaction effect	Mixed ANOVA: Time × Dosage interaction: participants over 2 years showed a decrease in K6 scores
			Regression: a significantly decreased level of K6 for the participants with 2-year intervention dosage compared to the comparison group with no intervention
	CSS Home Support	Significant	Time effect for both intervention and comparison groups. Dosage effect: a non-significant positive trend.

Although a majority of students in the intervention group showed decreases in K6 scores, there was a non-significant increase in K6 scores among the subsamples with no SFL participation and with 1-year SFL participation. The findings suggest that timing of data-gathering possibly influenced scores: post-program measurement was late in the school term and later in the year, when informal observation and teacher reports indicate higher levels of stress at school. Further investigation with the larger study will need to address such potential confounds. Nevertheless, the findings may point to a trend toward increasing levels of psychological distress during the adolescent developmental period for the participant group in this high-risk community (Figure 6). Further investigation with controls would be needed to confirm whether a moderating longer-term influence on psychological distress is associated with exposure to the program over 2 years, over and above changes associated with normal development (Figure 5). This would help to establish whether a classroom-taught curriculum offered during a critical developmental transition may have a preventive effect over time.

Neither prosocial behavior as measured by the SDQ nor self-concept as measured by CSS significantly changed in Study 1. As suggested, the underlying construct of the SDQ scale may not capture important dimensions of strength or relatedness relevant for this population. However, the results may suggest that the self-concept of adolescent students and aspects of social orientation to peers remain stable over time (36).

Students' perceptions of Home support showed mixed results (Figures 7, 8). While students with higher attendance at the SFL program retained higher perceptions of Home support, students with lower attendance showed an increasing trend in perceived Home support. These findings at least partially point to the conclusion that connectedness is a relevant dimension of external supports for well-being, and that absence of home support potentially mediates the distress experienced by many students in this population.

### Adverse Effects

Although levels of psychological distress as measured by the K6 appear to be very high (>18) for over a quarter of students in both study samples (Figure 6), there were no indications of adverse response to the program, or of acute episodes of individual distress beyond the range described. Teachers did not report any instances of adverse reaction to program subject matter, activities, or teaching methods. The school psychologist, who was responsible for monitoring at risk students and who regularly consulted with the teachers who taught SFL, did not identify any trend toward withdrawal or amplification of difficulty among students.

### Limitations

The small sample size and mixed intervention status of participating classrooms precludes fuller examination of psychometric properties of instruments used in this preliminary study. Tests of reliability and analyses of change scores were primarily conducted to inform decisions about the suitability of the scales for the future evaluation of the program. The comparison groups used in Study 2 were not randomly selected, so that attribution of changes to the effect of the program cannot be asserted. Further, small numbers do not permit analysis of differences between students according to the ability to self-complete the questionnaires and thus assessment of the effect of the mode of administration on consistency of responses. The variability in Cronbach's alpha coefficients suggests not only that careful choice of measures is needed but that development of purpose designed formats, response scales and modes of administration would be justified for this population.

## Conclusions

The results of this study highlight the complexity of local knowledge needed to establish appropriate processes of engagement and to inform decision-making about evaluation design in a challenging remote community setting. This kind of information is under-reported and for the most part not available in standard reviews of instruments and measures. As has been found elsewhere, careful attention must be paid to understanding of context in terms of participants' language and literacy levels, engagement with education, exposure to specific risks in their social context as well as the perspectives of knowledgeable practitioners when determining the appropriateness of measures for Indigenous populations (37). Findings of this study highlight the particular importance of such prior testing of measures with the target population before and during program development.

This preliminary pilot project confirmed the feasibility of the method of data-gathering and the potential utility of outcome measures based on student self-report, administered by structured interview in a remote school. To that extent they confirmed the evaluability of the program using these methods. However, not all measures trialed were suitable for continuing evaluation of SFL.

The analysis raised questions about the utility of the SDQ youth self-report version with this study population, in terms of reliability and validity of the scale and its alignment with program objectives as administered in this setting. Its performance was well-below that achieved in normative data for the general Australian population. Although in many studies referred to as a measure of resilience, the SDQ-SR does not appear to capture relevant dimensions of resilience, strengths, or difficulties targeted by the SFL program in the cultural context of remote Indigenous communities.

With this sample, the K6 scale showed higher internal consistency over all measurement points than the SDQ scales and appears suitable for continuing use for evaluation purposes. However, reliabilities were not consistently high, suggesting the need to consider additional, complementary measures of psychological well-being or distress. Nevertheless, findings pointed to a high burden of psychological distress in the student population, suggesting the need for investigation of stressors to which these students are exposed and to which they adapt in everyday life.

Despite its mixed results, the purpose-designed CSS scale confirmed that family and community resources, “external assets” that support resilience are important for Indigenous middle school students who experience multiple challenges and for whom the relationships and resources of family, school, and community may be essential for their ability to cope.

This pilot study provided important information to inform decision-making about the evaluation strategy for a preventative intervention in a remote middle school, and formed an essential stage of its development. Preliminary modeling of pre- and post-program changes provided some empirical evidence supporting the rationale for a resilience-building intervention with this student population and for the evaluability of potential benefits. Together with the process of practitioner review, these confirmed an absence of iatrogenic effects in a population subject to a high burden of psychological distress. The findings of this preliminary pilot suggest that an evaluation framework for social-emotional learning in a remote population needs to be sensitive to changes in individual resilience and competencies, in levels of psychological distress, and in students' perceptions of external socio-cultural resources and supports that assist in coping with multiple developmental challenges and life stressors.

## Data Availability Statement

The datasets presented in this article are not readily available because according to conditions of ethics approval, identification of data by individual, classroom/year, and location is not permitted without individual consent; data are not publicly available. Limited access may be considered on request. Requests to access the datasets should be directed to gary.robinson@menzies.edu.au.

## Ethics Statement

The studies involving human participants were reviewed and approved by Human Research Ethics Committee of the Northern Territory Department of Health and the Menzies School of Health Research (HREC 2013-2120). Written informed consent to participate in the study was obtained from the participants' legal guardian/next of kin.

## Author Contributions

GR was project leader, responsible for concept development, implementation of intervention program in schools, oversight of the evaluation project, and first drafting of Discussion and Conclusions. EL was chief statistician responsible for all data analysis for this paper. GR, SS, PN, and RM were chief investigators responsible for design of the program evaluation, including choice of measures and methods, and contributed to interpretation of findings, drafting, and revision of the paper. BL was evaluation project coordinator 2014–2018, oversaw data-gathering and data management, ethics and procedures, and initial analysis and contributed to evaluation design, interpretation of findings, and conclusions. All authors contributed to the article and approved the submitted version.

## Conflict of Interest

The authors declare that the research was conducted in the absence of any commercial or financial relationships that could be construed as a potential conflict of interest.
